# Expectancy Violations and Discontinuance Behavior in Live-Streaming Commerce: Exploring Human Interactions with Virtual Streamers

**DOI:** 10.3390/bs14100920

**Published:** 2024-10-09

**Authors:** Yanhong Chen, Xiangxia Li

**Affiliations:** School of Information Science, Guangdong University of Finance and Economics, Guangzhou 510320, China

**Keywords:** live-streaming commerce, virtual streamers, expectancy violation, discontinuance behavior, distrust, dissatisfaction

## Abstract

Virtual streamers, as a typical application of AI-enabled digital humans, are increasingly being utilized in live-streaming commerce due to technological advancements and industry innovations. Although virtual streamers present several benefits, there is potential for adverse effects when they do not align with consumer expectations. Drawing upon expectancy violations theory, this study developed a theoretical model to explore whether and how consumers’ expectation violations during human–virtual streamer interactions affect consumers’ discontinuance behavior. Through an online questionnaire survey of 307 Chinese consumers with prior experience interacting with virtual streamers, this study used a partial least squares structural equation model to analyze the research model. The empirical results indicated that professionalism expectation violation, empathy expectation violation, and responsiveness expectation violation positively influenced consumers’ distrust and dissatisfaction, which subsequently led to discontinuance behavior. This study contributes to the literature on live-streaming commerce, human–AI interaction, and expectancy violation theory. Furthermore, the findings offer valuable insights for practitioners in the field of live-streaming commerce by enabling them to formulate preventive or remedial strategies to mitigate potential negative outcomes when implementing virtual streamers.

## 1. Introduction

E-commerce has utilized virtual streamers for live streaming to optimize cost-efficiency and provide round-the-clock services to consumers, with unprecedented growth expected in the near future [1]. According to iiMedia Research [2], nearly 90% of the respondents expressed their intention to make purchases through virtual streamers, and 47.5% of consumers were highly optimistic about the prospects of virtual live-streaming commerce in China. Virtual streamers, also referred to as digital or AI streamers, are computer-generated and AI-powered characters that possess a human-like appearance [3]. They are designed to mimic human-like features, possessing the social interaction abilities to facilitate transactions and enhance the overall experience in live-streaming commerce [4]. Compared with human streamers, virtual streamers are recognized for their consistency, scalability, and cost-effectiveness [5].

However, despite the technical advances, the practical application of virtual streamers has yet to achieve satisfactory performance and frequently fails to meet consumer expectations [5,6]. For instance, virtual streamers may provide unsuitable responses to consumer requests, lack genuine human emotion, and offer limited information to consumers [6]. These issues may significantly undermine consumers’ shopping experiences, resulting in negative outcomes, such as reluctance to use virtual streamer services or negative word of mouth [7]. The study of Gao et al. [7] suggests that AI streamers lack perceived intimacy and responsiveness compared with human streamers, thus diminishing consumers’ purchase intention. Therefore, it is crucial to understand and avoid the potential negative consumer behavior that may arise during human–virtual streamer interactions. Previous studies on virtual streamers have revealed the advantages of virtual streamers with anthropomorphic traits [8,9], warmth factors [1], and sociability [4] in bolstering a consumer’s trust, engagement, experiential value, and purchase intention. Although these studies have mostly reported the factors affecting the effectiveness of virtual streamers, few have paid attention to the unpleasant experiences, consequences, and mechanisms that arise when consumers encounter poor performance from virtual streamers.

To bridge this gap, we draw on expectation violation theory (EVT) and computers as social actors (CASA) theory to explore the negative outcomes of consumers’ interactions with virtual streamers, an aspect that has been less discussed in prior research. According to CASA theory, people tend to treat human-like virtual streamers as social actors and behave socially in interactions with them [10,11]. When entering a live broadcast room, the consumer first notices the appearance of a virtual streamer before engaging in formal interaction. The initial impression of the virtual streamers directly influences consumers’ subsequent expectations [12]. Consumers may perceive anthropomorphic virtual streamers as having the same efficiency and competencies as human streamers [13,14]. Miyan Liao et al. [15] pointed out that the competencies of human streamers encompass cognitive, emotional, and social dimensions, which play a crucial role in attracting a substantial viewership and driving live-streaming sales. Therefore, based on CASA theory and the work of Miyan Liao et al. [15], we propose that consumers in the initial stage of interacting with virtual streamers may expect them to possess the same cognitive, emotional, and social competence as human streamers.

Nevertheless, if the actual performance of virtual streamers fails to meet consumer expectations regarding their competencies, negative outcomes may occur as the interaction deepens [5]. Previous research has demonstrated the positive effect of streamers’ competencies on consumers’ experiences [1,7]. However, the consequences of a lack of these competencies (or being lower than expected) on consumers’ negative behavior in the virtual live-streaming context have remained underexplored. Therefore, we draw on EVT to explain the negative outcomes when consumers’ expectations are violated while interacting with virtual streamers. Specifically, based on the “expectation violations–psychological state–behavior outcome” framework, we examined the mediation effect of distrust and dissatisfaction on the relationships between competency expectation violations and consumers’ discontinuance behavior. Two research questions are addressed in this study: (1) How do expectation violations affect consumers’ distrust and dissatisfaction while interacting with virtual streamers? (2) How do distrust and dissatisfaction with virtual streamers influence consumers’ negative behavior?

Our study contributes to the existing literature in several ways. First, existing research has extensively studied human streamers in live-streaming commerce, while the attention given to virtual live streamers remains limited. By drawing on EVT, this study focuses on virtual streamers in the context of live-streaming commerce and examines the underlying mechanism for negative user behaviors resulting from expectation violations. Thus, this study not only enriches the live-streaming commerce research involving virtual streamers, but also extends the EVT in novel fields. Second, this study contributes to human–AI interaction by exploring the negative consequences of expectation violation regarding human-like competencies. Specifically, we conceptualize and decompose the expectation violations into three dimensions: cognitive (professionalism expectation violation), emotional (empathy expectation violation), and social (responsiveness expectation violation). We then uncover how these violations influence consumers’ feelings of distrust and dissatisfaction, leading to discontinuance behavior. Third, this study advances the literature by revealing that the distrust and dissatisfaction with virtual streamers mediate the effect of expectation violations on discontinuance behavior. From a practical perspective, this study encourages live-streaming practitioners and information technology managers to appropriately adjust the implementation strategies of virtual streamers according to the three dimensions of expectation violations.

The next section presents a literature review on virtual streamers in live-streaming commerce and expectancy violation theory. Section 3 presents our research model and discusses the corresponding hypotheses. Section 4 describes the methodology and research design. The analysis results are presented in Section 5. In Section 6, we discuss the implications for both research and practice and the limitations of this study.

## 2. Literature Review and Theoretical Background

### 2.1. Virtual AI Entities and Virtual Streamers in Live-Streaming Commerce

The advancement of AI has enabled the fabrication of virtual entities, such as avatars, digital assistants, virtual influencers, humanoid chatbots, and virtual agents, for use in services and marketing [13,16]. These virtual AI entities are characterized by their remarkable ability to mimic human behaviors, often featuring life-like appearances, natural language-processing capabilities, and the capacity to learn and adapt to user preferences and behaviors over time [17]. Previous research has examined the performance and impact of virtual AI entities in different contexts and analyzed individuals’ perceptions and feelings during human–AI interaction from various perspectives [18]. For instance, Ashfaq et al. [19] investigated the impact of human–chatbot interaction quality on users’ satisfaction and continuance intention. Moussawi et al. [20] examined how users’ perceptions of personal intelligent agents’ characteristics (e.g., intelligence, anthropomorphism, and self-extension) influence their post-adoption evaluations. Zhang et al. [21] argued that response speed, response accuracy, convenience, and accessibility positively influence service quality in the e-commerce customer service context. These studies have examined how individuals perceive and interact with virtual AI entities, as well as how these interactions shape their overall experience, adoption, and post-adoption behavior. Therefore, the current research on virtual AI entities provides valuable insights into human–AI interactions and enhances our understanding of individuals’ experiences and behaviors in human interactions with virtual streamers.

As a significant application for virtual AI entities, virtual streamers are “digital avatars with anthropomorphic appearances that are able to emulate human streamers” [22] (p. 2). These digital avatars are often equipped with AI attributes, such as voice, speech, expression, and gesture, enabling them to establish a unique presence within the domain of live-streaming commerce [4]. As AI-generated personas, virtual streamers are designed to mimic human-like features and behaviors, enabling them to provide live-streaming services comparable to those of real people and create an engaging and interactive experience for consumers [3].

Previous studies have examined individuals’ perceptions and experiences of interacting with virtual streamers from different perspectives, such as virtual streamer characteristics [1], technical features [9], socialness [4], and linguistic style [3]. For instance, Gao et al. [1] discovered that a virtual streamer’s characteristics (i.e., likeability, animacy, and responsiveness) positively influence purchase intention by enhancing social presence and telepresence. Yuan Sun et al. [9] highlighted that the technical features of virtual streamers, including anthropomorphism and media richness, affect customers’ purchase intention, with this effect being mediated by psychological distance and customer engagement. In general, the existing research mainly discusses the positive effects of virtual streamers on consumers’ experience and behaviors, as well as examines the performance differences between virtual and human streamers [1,9,23,24]. However, some scholars have found that virtual streamers do not always lead to positive consequences [5]. Hu and Ma [22] discovered that virtual streamers’ use of sensory language can lead to a decrease in purchase intention. Peng et al. [6] proposed that AI-oriented live-streaming commerce service failures result in consumers’ disappointment and emotional exhaustion. Furthermore, the relevant research on virtual streamers focused more on initial adoption than on post-adoption behavior. Therefore, by proposing a framework that investigates the post-adoption behavior arising from expectation violations, our study explores the negative consequences of human–virtual streamer interactions in the live-streaming commerce context.

### 2.2. CASA Theory Applied to Virtual Streamers: Consumers’ Expectations of Virtual Streamers’ Competencies

The computers as social actors (CASA) theory posits that humans tend to mindlessly perceive computers and machines as social actors when they are provided with sufficient social cues, leading them to apply human norms and behaviors in their interactions with these technological entities [25,26]. These reactions are often elicited by social cues, such as language use, the human voice, and human-like facial features conveyed by technology [4]. Given the ongoing AI revolution, CASA theory has been applied to various AI-oriented services, such as digital humans, chatbots, social robots, and virtual agents [26]. Previous research conducted within the CASA framework demonstrated that individuals exhibit social responses when interacting with anthropomorphic agents [17,27,28]. Thus, CASA theory provides a basis for understanding consumers’ reactions and expectations while interacting with virtual streamers.

In the context of live-streaming commerce, virtual streamers often exhibit human-like physical appearances as well as rich expressions and vocalizations [8]. When a virtual streamer displays these social cues, consumers unconsciously treat them as social actors and apply human–human streamer communication norms to human–virtual streamer interactions [3,4]. The existing research on live-streaming commerce highlights the importance of streamers’ competencies, emphasizing that streamers’ competencies play a pivotal role in optimizing consumer experiences and influencing their purchasing decisions [1,15]. Previous research has shown that consumers have human-like competency expectations toward chatbots [12,29]. Therefore, it is likely that consumers expect a congruence in competencies between human streamers and anthropomorphic digital streamers in live-streaming commerce. They may expect virtual streamers to be capable of generating a high level of human-like competencies and respond to them in a manner akin to their interactions with human streamers [29].

According to Miyan Liao et al. [15], the competencies of real human streamers encompass cognitive, emotional, and social dimensions. We believe that consumers also have similar expectations for virtual streamers’ competencies. Thus, we infer that consumers also expect virtual streamers to possess cognitive, emotional, and social competencies [29]. Specifically, cognitive competency represents the professional ability that a streamer possesses related to the knowledge of live product attributes [15,30]. When engaging with virtual streamers, consumers expect to receive accurate information and product guidance to reduce their efforts in product search and evaluation [31]. The cognitive competency is mainly reflected in the professionalism exhibited by virtual streamers [15]. Emotional competency is related to the streamers’ ability to recognize, understand, and express emotions [29,32]. Consumers expect virtual streamers to display human-like warmth and compassion during interactions to satisfy their emotional needs [3]. Empathy is a key dimension of emotional competency and serves as a prominent signal through which individuals can effectively convey their understanding of others’ emotions [32]. Social competency refers to the intensity and effectiveness of streamers’ responses to consumers’ needs and concerns [15,33]. We identified responsiveness as a pivotal facet of social competency, which reflects virtual streamers’ ability to effectively and promptly interact with consumers.

### 2.3. Expectancy Violations Theory

Expectancy violations theory (EVT), originally formulated to explain expectations related to communication [34], has since been expanded to encompass various violations in human or non-human verbal or non-verbal behaviors [11,35]. EVT assumes that people always hold expectations in the interaction process [36]. When these expectations are violated, whether perceived positively or negatively, individuals tend to pay special attention to the underlying reasons for the violation and make efforts to alleviate the resulting cognitive dissonance [37]. Scholars have applied EVT to study human–computer and human–AI interactions [12,36,37]. For instance, Zhou et al. [37] extended the EVT to short-form video game platforms and found that users’ gamification interaction expectation violations could positively impact users’ psychological experience and result in negative user behavior. Crolic et al. [12] applied the EVT to study chatbot-driven customer service and found that anthropomorphism increases customers’ expectations about a chatbot’s performance capabilities, resulting in expectancy violations. However, there is a lack of research on the application of EVT in the context of virtual streamers’ live streaming. Thus, this study attempts to apply the EVT to explore how expectation violations pertaining to virtual streamers’ competencies affect consumers’ experiences and behaviors.

According to CASA theory, the initial interaction with virtual streamers makes consumers perceive them as social actors and expect them to possess the same competencies as human streamers. When the performance of virtual streamers fails to meet consumers’ expectations, expectation violations occur. Specifically, we identified consumers’ expectation violations regarding virtual streamers’ competencies in three aspects: professionalism expectation violation (PEV), empathy expectation violation (EEV), and responsiveness expectation violation (REV). Consumers expect virtual streamers to be as capable as real human streamers; however, compared to human streamers, virtual streamers have limitations in addressing complex problems, providing effective responses, and exhibiting empathy [7]. Consequently, this discrepancy may result in expectation violations and adversely impact consumer behavior.

## 3. Research Model and Hypotheses

**Figure 1:**
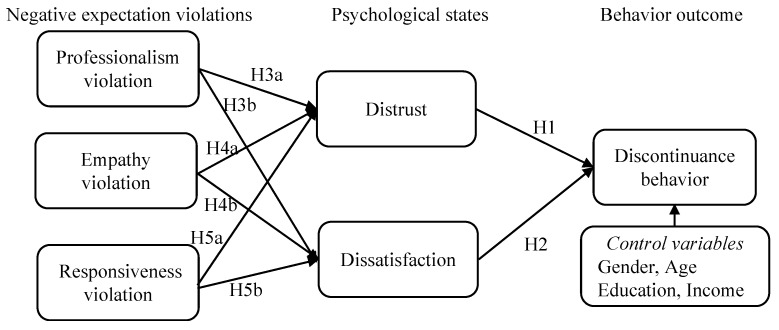
Research model.

Based on the above literature review, we developed our research model, as presented in Figure 1. Drawing upon expectation violation theory and the “expectation violations–psychological states–behavior outcome” framework, we attempted to explain the mechanism of the negative outcomes of expectation violations in the virtual live-streaming commerce context. CASA theory posits that consumers will interact with virtual streamers with certain expectations due to their anthropomorphism [10]. However, when consumers’ perceptions fail to align with their expectations, i.e., negative expectation violations occur, their affective states will be negatively impacted, resulting in distrust and dissatisfaction with virtual streamers [11]. Subsequently, these negative affective states exert an influence on consumers’ behavior.

In addition, prior research has suggested that demographic factors, such as gender, age, education, and income, may affect consumers’ adoption and post-adoption behaviors [9,19,38]. For instance, Huang and Yu [39] revealed differences between men and women in terms of their continuance intention to watch AI news anchors. Yuguang Xie et al. [40] showed that younger users were more likely than older users to adopt AI assistants. Therefore, we included demographic factors as control variables to examine their potential influence on consumers’ discontinuance behavior.

### 3.1. Distrust, Dissatisfaction, and Discontinuance Behavior

In the context of live-streaming commerce, discontinuance behavior can be defined as individuals reducing their level of engagement with virtual streamers or temporarily or permanently ceasing to watch them [41,42].

Distrusting belief is defined as “the degree to which one believes, with feelings of relative certainty, that the other person or entity does not have characteristics beneficial to one” [43] (p. 44). Previous research has argued that individual distrust belief can lead to distrust-related behaviors, such as a decrease in the intensity of information system usage [44], resistance to AI chatbots [32], and discontinuance intention of WhatsApp [45]. In the context of this study, when consumers develop mistrust toward virtual streamers, it can result in diminished confidence in virtual streamers’ capabilities, leading to decreased engagement and a lower likelihood of continued watching. Accordingly, we propose that:

**H1.**  *Distrust in virtual streamers is positively related to discontinuance behavior.*

Dissatisfaction reflects negative feelings, such as irritation or frustration, that individuals may have toward their overall experience with a product or service [41,46]. Dissatisfaction with virtual streamers, in particular, indicates that consumers feel discontented after engaging with virtual streamers. Previous research has found that such negative feelings would lead to unfavorable consumer behaviors, such as discontinuing usage, restricting use, or switching to alternatives [41,47]. For example, Zhang et al. [41] found a positive relationship between dissatisfaction and discontinuance intention. Therefore, consumers who experience negative feelings toward virtual streamers are less likely to invest time and effort in engaging with them in the future. Instead, they may seek real human streamers as a substitute for virtual ones. Hence, we hypothesize the following:

**H2.**  *Dissatisfaction with virtual streamers is positively related to discontinuance behavior.*

### 3.2. Negative Expectation Violations and Distrust in Virtual Streamers

According to Darke et al. [48], when negative expectation violation occurs, it leads to customer distrust. Consumers evaluate their experience of watching virtual streamers and ultimately assess the knowledge, effectiveness, and intelligence of these virtual streamers. If virtual streamers fail to meet consumers’ expectations, this may result in an amplifying or boomerang effect, ultimately leading to a level of distrust in them [49]. The impacts of PEV, EEV, and REV on distrust are formulated as follows.

The level of professionalism of streamers reflects their capability to effectively communicate appropriate knowledge, experience, and skills to consumers [50,51]. Empirical research on real human streamers has shown that professional streamers engender greater trust among viewers [31,52]. Individuals often perceive AI-based entities as having advanced cognitive capabilities, thus setting high expectations regarding the quality and accuracy of live content from virtual streamers [6,53,54]. When virtual streamers perform poorly in demonstrating their professionalism, such as lacking essential product knowledge, frequently making factual errors, or providing inaccurate information, it can lead consumers to doubt their ability and reduce trust in them [32]. Therefore, we hypothesize the following:

**H3a:**  *Professionalism expectation violation (PEV) is positively related to distrust in virtual streamers.*

Empathy refers to streamers’ capacity to sense and react to an individual’s thoughts, feelings, and experiences [32]. This capacity is crucial for streamers as it enables them to demonstrate personalized care and attention toward their viewers [55]. Empathy has been shown to play a central role in the trust-building process [32,56], whereas its absence can lead to distrust. For example, Yang et al. [32] found that the absence of empathy can increase distrust in AI-based customer service. Consumers generally expect a certain level of emotional intelligence and understanding from virtual streamers [11,57]. If these expectations are not met, particularly in moments where consumers seek emotional support or validation, it can lead consumers to feel detached and unimportant, thereby increasing their distrust in virtual streamers. Hence, we hypothesize the following:

**H4a:**  *Empathy expectation violation (EEV) is positively related to distrust in virtual streamers.*

Responsiveness reflects streamers’ ability to assist consumers and deliver timely service [58,59]. Previous studies have shown that the immediacy of responses from real human streamers helps to form a relational bond between consumers and streamers, thus enhancing trust in streamers [33,59]. On the other hand, slow responsiveness and inefficient communication may hinder consumers’ trust [60]. In this study, individuals who engage with virtual streamers expect to receive fast and effective replies from them [1]. When consumers encounter delayed responses or feel ignored by virtual streamers, they may perceive them as less capable and efficient in problem resolution. Therefore, failure to address consumer issues in a timely manner may damage consumer trust in virtual streamers and subsequently lead to distrust in them. Hence, we propose the following:

**H5a:**  *Responsiveness expectation violation (REV) is positively related to distrust in virtual streamers.*

### 3.3. Negative Expectation Violations and Dissatisfaction with Virtual Streamers

According to the expectancy disconfirmation theory, when the actual performance of a product or service fails to meet consumers’ expectations, it will lead to consumer dissatisfaction [61]. Previous studies have extensively demonstrated the impact of negative expectancy violations on consumer dissatisfaction [62,63,64]. The effects of PEV, EEV, and REV on dissatisfaction are formulated as follows.

Previous studies have indicated that the information provided by streamers with high professionalism greatly reduces consumers’ costs in product search and evaluation, and notably enhances their satisfaction during the purchasing process [15,30]. Drawing upon the expectation disconfirmation theory, if consumers’ expectations regarding the credibility, accuracy, or professionalism of virtual streamers are not met—for instance, when a virtual streamer provides inaccurate information or engages in misleading behaviors—consumers might experience dissatisfaction with the virtual streamer [64]. Corroborating this notion, Peng et al. [6] discerned that when the information quality of AI-oriented live streaming goes against consumers’ expectations, it could result in consumer dissatisfaction. Therefore, we propose the following hypothesis:

**H3b:**  *Professionalism expectation violation (PEV) is positively related to dissatisfaction with virtual streamers.*

Existing literature on live-streaming commerce has demonstrated that the empathy shown by real human streamers during interactions and their concern for consumers’ needs and interests make consumers feel more comfortable and satisfied [3,59]. Furthermore, research on human–AI interactions has indicated that empathy improves satisfaction in service robots and AI devices [32,65]. Therefore, similar to empathetic human streamers who can increase the satisfaction and relationship quality between consumers and streamers, the presence of empathy in virtual streamers can increase consumers’ trust and satisfaction toward them. According to CASA theory [65], consumers expect virtual streamers to be empathetic (similar to human streamers). If virtual streamers fail to sense and react to a consumer’s thoughts, feelings, and experiences, it often leads to a sense of frustration and disappointment in the consumer [6], thereby fostering dissatisfaction. Thus, we hypothesize the following:

**H4b:**  *Empathy expectation violation (EEV) is positively related to dissatisfaction with virtual streamers.*

The relationship between a lack of responsiveness and dissatisfaction has been examined in relationship marketing and the service industry [66]. Specifically, Mostafa et al. [64] concluded that the negative parasocial interaction disconfirmation of virtual agents, such as failing to respond suitably to consumers’ requests, is positively associated with dissatisfaction with virtual agents. Responsiveness emphasizes the streamers’ capacity to provide instant responses [1]. This quick response service can diminish the psychological distance between consumers and streamers, enhancing their connection and promoting consumer comfort and satisfaction with their interaction experience [58]. In contrast, the absence of responsiveness can engender a sense of neglect or disregard among consumers, resulting in a perception of an inefficient and unfriendly shopping process, ultimately leading to dissatisfaction [6]. Therefore, we propose the following hypothesis:

**H5b:**  *Responsiveness expectation violation (REV) is positively related to dissatisfaction with virtual streamers.*

## 4. Research Methodology

### 4.1. Measurement Items

All the measurement items for each latent construct were adapted from previous research and modified to suit the context of virtual streamers’ live-streaming commerce. Table 1 provides definitions of the variables, measurement items, and key related literature. Specifically, items for PEV were adapted from Li et al. [50] and Guo et al. [30]. Items for EEV were adapted from Yang et al. [32]. Items for REV were adapted from Gao et al. [1]. Items for dissatisfaction were adapted from Mostafa et al. [64], and items for distrust were adapted from Yang et al. [32]. Items for discontinuance behavior were adapted from Peng et al. [6]. These measurement items were evaluated using a 7-point Likert scale, ranging from 1 (strongly disagree) to 7 (strongly agree).

### 4.2. Research Design and Data Collection

To empirically test our hypotheses, we collected data using online questionnaires distributed via Credamo.com, a platform in China that is equivalent to Amazon’s Mechanical Turk and is known for its efficient survey services. With over three million registered users and its high efficiency in data collection, Credamo has been widely used by scholars to distribute questionnaires [67,68]. We initially sent questionnaires to experts in the human–AI interaction research field and sought their advice regarding the clarity and ambiguity of the measurement items. According to their suggestions, we refined and finalized the questionnaire, which consisted of three sections. The first section included an introductory consent form for participants, which informed them about the study’s purpose, their voluntary and anonymous participation, the scope of the data usage, and the protection of their personal data. All surveys were conducted with the participants’ consent, and they had the option to withdraw from the survey at any time. The second section began with a screening question to filter participants based on their experience with virtual streamers, allowing only those who answered “yes” to proceed with the questionnaire. Then, the qualified participants responded to the inquiry on the research constructs based on their perceptions and experiences of virtual streamers in live-streaming commerce. The third section collected demographic information from participants, including gender, age, education level, and monthly income.

Finally, a total of 307 valid questionnaires were obtained. The characteristics of the respondents are presented in Table 2. Of the total sample, 45.9% were male and 54.1% were female. The majority of respondents (78.1%) were aged below 35 years. Roughly 96% of the respondents possessed at least a college degree. More than half of the participants (66.7%) reported a monthly income exceeding 6000 yuan.

## 5. Data Analysis and Results

The partial least squares structural equation modeling (PLS-SEM) approach was adopted to analyze the measurement and structural model. PLS-SEM was chosen because it is more suitable for theory exploration and prediction compared to the covariance-based structural equation modeling (CB-SEM) method [69,70]. We not only tested existing theories but also expanded the theoretical framework by adding new paths and extending into a new research context. Additionally, PLS-SEM is not strict regarding residual distributions and sample size, allowing researchers to examine samples smaller than 500 [71]. We obtained a total of 307 usable responses, which exceeds the minimum requirements [69] but may result in non-normal data distributions due to the convenience sampling method. Thus, PLS-SEM was deemed an appropriate choice for this study. For conducting model analysis, we utilized the Smart PLS 4.0.

### 5.1. Common Method Bias and Multicollinearity Test

Since our study relied on self-reported survey data, there was a potential risk of common method bias (CMB). Following the approach recommended by Liang et al. [72], we conducted a statistical analysis by incorporating a common method factor that encompassed all items as indicators. The results, presented in Table 3, indicate that the average substantively explained variance of the indicators was 0.747, whereas the average method-based variance of the indicators was 0.008. The ratio between them was approximately 88:1. Moreover, most of the method factor loadings were found to be insignificant. As indicated by the small magnitude and insignificant results for method variance, common method bias was not a significant issue in our study. Additionally, we also tested for the existence of multicollinearity in the model. As shown in Table 4, all variance inflation factor (VIF) values were below 3.3 [73], indicating that multicollinearity was unlikely to be a concern in this study.

### 5.2. Measurement Model

We employed Cronbach’s alpha and composite reliability (CR) to test the internal consistency of the constructs’ measurements. As presented in Table 4, both Cronbach’s α (ranging from 0.849 to 0.886) and CR (ranging from 0.855 to 0.887) for each construct exceeded the threshold value of 0.7, and all item loadings exhibited significance and exceeded a value of 0.7. Furthermore, the average variance extracted (AVE) for each construct was above 0.5. Consequently, these results demonstrated a satisfactory level of reliability and convergent validity.

Table 5 shows the results of the Fornell–Larcker criterion and hetero-trait–mono-trait ratio of correlations (HTMT). As shown in Table 5, the diagonal elements display the square root of the AVE for each construct, which was higher than the inter-correlations among constructs, demonstrating acceptable discriminant validity according to the Fornell–Larcker criterion. In addition, the HTMT ratio of the correlations exhibited all values below the threshold of 0.85 [74], thereby providing further support for discriminant validity.

### 5.3. Structural Model

**Figure 2:**
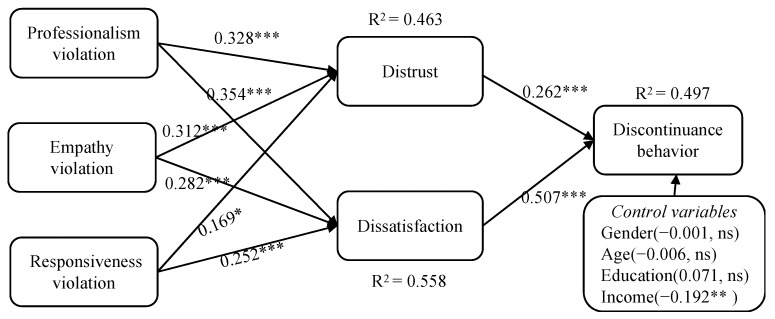
PLS results. Note: *** *p* < 0.001, ** *p* < 0.01, * *p* < 0.05, ns: not significant.

A bootstrapping technique with a resample size of 5000 was employed to test our hypotheses. The results of path coefficients and R^2^ values for each endogenous latent variable are presented in Figure 2. In general, all hypotheses were supported. Moreover, the variance explanation proportions (R^2^) for discontinuance behavior, distrust, and dissatisfaction were 49.7%, 46.3%, and 55.8%, respectively. The Q^2^ values for discontinuance behavior (0.378), distrust (0.346), and dissatisfaction (0.408) were all above 0.15, indicating sufficient predictive relevance for these endogenous variables [69]. Additionally, we adopted the standardized root mean square residual (SRMR) and the Normed Fit Index (NFI) to assess the model fit. The SRMR value was 0.059, which is below the 0.08 threshold, and the NFI was 0.861 (>0.800), indicating the model fit acceptably [75]. Regarding the control variables, the analysis results showed that only monthly income (β = −0.192, *p* < 0.01) significantly affected discontinuance behavior, while gender, age, and education had no significant effects.

### 5.4. Mediation Effects of Dissatisfaction and Distrust

To verify whether distrust and dissatisfaction mediated the relationship between expectation violations (PEV, EEV, and REV) and discontinuance behavior, a two-tailed test and bias-corrected accelerated bootstrapping were conducted in Smart PLS 4.0 [75]. The results, shown in Table 6, suggest that all of the indirect effects were significant, with the 95% confidence interval excluding zero. Distrust was found to significantly mediate the effects of PEV (β = 0.086, *p* < 0.01), EEV (β = 0.082, *p* < 0.01), and REV (β = 0.044, *p* < 0.05) on discontinuance behavior. Similarly, dissatisfaction was found to significantly mediate the effects of PEV (β = 0.180, *p* < 0.001), EEV (β = 0.143, *p* < 0.001), and REV (β = 0.128, *p* < 0.001) on discontinuance behavior. Taken together, these results suggest that both distrust and dissatisfaction mediated the relationship between PEV, EEV, REV, and discontinuance behavior.

## 6. Discussion and Implications

### 6.1. Discussion of the Results

Despite the majority of research on live-streaming commerce focusing on human streamers, there is limited knowledge about virtual streamers. This study sought to explore the adverse consequences arising from negative expectation violations when consumers interact with virtual streamers. In particular, this research investigated how PEV, EEV, and REV impact consumers’ discontinuance behavior, mediated by distrust and dissatisfaction. Through this study, we argue that live-streaming practitioners and information technology managers should take proactive steps to meet or exceed consumer expectations of virtual streamer competencies in order to mitigate potential negative outcomes.

First, both distrust (β = 0.262, *p* < 0.001) and dissatisfaction (β = 0.507, *p* < 0.001) were found to have a positive association with discontinuance behavior, thus supporting H1 and H2. This supports the previous research conducted by Yang et al. [32], which showed that customer distrust in chatbots reduces their resistance to them. Additionally, this also aligns with prior research on AI-based entities, such as chatbots and virtual agents, which concluded that satisfaction is a vital determinant of intention to reuse [60,64]. In addition, this study revealed that dissatisfaction had a stronger impact on discontinuance behavior compared to distrust. Our study has extended the understanding of the relationship between distrust/dissatisfaction and discontinuance behavior in the novel context of human–virtual streamer interactions.

Second, the empirical results verified that the violations of professionalism (β = 0.328, *p* < 0.001), empathy (β = 0.312, *p* < 0.001), and responsiveness (β = 0.169, *p* < 0.05) were positively associated with distrust, thus supporting H3a, H4a, and H5a. These findings are consistent with those of Yang et al. [32], which showed that the drawbacks of AI-based chatbots, such as a lack of empathy and the provision of irrelevant or biased information, can lead to user distrust. The study results suggested that if consumers perceive virtual streamers as lacking the expected communication abilities, such as understanding, empathy, and responsiveness, that consumers anticipate, it may lead to an increase in distrust in virtual streamers.

Third, the findings demonstrated that the violations of professionalism (β = 0.354, *p* < 0.001), empathy (β = 0.282, *p* < 0.001), and responsiveness (β = 0.252, *p* < 0.05) were positively associated with dissatisfaction, thus supporting H3b, H4b, and H5b. The findings revealed that when virtual streamers fail to meet consumers’ expectations of human-like competencies, it can result in dissatisfaction with the virtual streamers. The findings align with previous research, which implied that customers’ interaction with AI-enabled non-human entities that go against their expectations may lead to dissatisfaction [6,64]. Our findings complement previous research (e.g., [29,53]), which emphasized the importance of human-like competencies for AI agents in influencing consumer satisfaction. Furthermore, the results align with expectation disconfirmation theory [61], demonstrating that consumers interact with virtual streamers with pre-determined expectations regarding their human-like competencies. If these expectations are not met, they tend to be dissatisfied with virtual streamers.

Finally, our study found that distrust and dissatisfaction mediated the relationship between the three aspects of expectation violations and discontinuance behavior, further validating the central role of affective factors in translating expectation violations into negative behaviors. Specifically, when consumers evaluate their experience of interacting with virtual streamers that fail to meet their expectations, their affective attitudes toward the virtual streamers will be negative, which in turn leads to discontinuance behavior. This result corresponds with previous studies [37,41,63], which highlighted the mediating effect of psychological states in the cognition–behavior nexus. This finding helps us understand the series of changes in individual psychological reactions when expectation violations occur in human–virtual streamers interactions. It underscores the importance of addressing consumer dissatisfaction and distrust as a key strategy for retaining consumer engagement and preventing discontinuance behavior in the virtual streaming context.

### 6.2. Theoretical Implications

Our study made several contributions to the literature. First, our findings extended the live-streaming commerce literature by delving into the potential negative effects when virtual streamers’ performance violates consumers’ expectations. Research on live-streaming commerce has largely focused on consumers’ positive perceptions of virtual streamers, such as increasing perceptions of social presence and telepresence [1], impacting individuals’ sense of engagement [9], and enhancing consumer brand forgiveness [24]. Drawing from CASA theory, we acknowledged that consumers engage with virtual streamers with certain expectations due to their anthropomorphism [10]. Contrary to prior studies that primarily emphasized the positive aspects of virtual streamers, our study proposed that discrepancies between consumers’ expectations and the actual performance of virtual streamers may lead to negative consequences, and we further explored the underlying mechanism behind it, thereby broadening the research boundaries of live-streaming commerce. Furthermore, our study contributed to the understanding of discontinuation behavior and the factors that induce such behavior in AI-enabled live-streaming commerce contexts.

Second, this study provided valuable insights into the literature on human–AI interactions by exploring the mechanisms of expectation violation regarding human-like competencies within the domain of virtual live-streaming commerce. Drawing upon the multidimensional nature of streamers’ competencies, as proposed by Miyan Liao et al. [15], this study offered insightful perspectives on the effects of expectation violation of human-like competencies (cognitive, emotional, and social) in virtual streamers. Our findings revealed that when virtual streamers are perceived as lacking human-like competencies, such as providing inadequate responses, lacking authentic emotion, or offering limited information to consumers, this can lead to consumer distrust and dissatisfaction toward the virtual streamers. Consequently, we underscored the importance of fostering consumers’ trust and satisfaction toward virtual streamers by meeting expectations about three human-like competencies in the context of human–virtual streamer interaction.

Third, this study applied the EVT to the field of live-streaming commerce and proposed three types of competencies’ expectation violations, namely, PEV, EEV, and REV. This expanded the scope of EVT, which was previously applied in conversational agents [35], chatbots [12], and AI speakers [76]. Further, this study investigated how these three violations led to consumers’ distrust and dissatisfaction with virtual streamers, ultimately resulting in discontinuance behavior. These findings contribute to the literature on negative perceptions and responses in the context of AI-enabled live-streaming commerce.

### 6.3. Practical Implications

This paper has several practical implications for live-streaming marketing personnel, as well as for managers in live-streaming platforms and information technology (IT). First, it is necessary to clearly inform consumers that they are interacting with AI-based streamers. By doing so, their expectations for the interaction can be adjusted to be lower than those typically held for human streamers [35]. For example, it should be explicitly stated in the live room that the streamer is AI-powered, and a concise explanation of its capabilities and limitations should be provided. Setting modest expectations for these virtual streamers allows consumers to be pleasantly surprised when their expectations are exceeded, rather than feeling frustrated when their expectations are unmet. This approach can benefit the company by reducing the instances of negative expectation violations that lead to consumer distrust and dissatisfaction when utilizing AI-powered streaming services.

Second, managers of IT and live-streaming platforms need to be keenly aware of the inherent limitations of virtual streamers in fully meeting consumers’ expectations. These limitations often stem from the technological and algorithmic constraints of AI, which may lead to unexpected behaviors or responses that violate consumers’ expectations. To mitigate these issues, IT managers should continuously optimize virtual streamers’ capabilities, adopting more advanced artificial intelligence and machine learning technologies that enable virtual streamers to better respond to consumers’ needs and preferences. They should train virtual streamers to enhance their ability to provide expert descriptions of products to target consumers, effectively address consumers’ requirements, and express empathy in communication with consumers. For example, virtual streamers should be programmed to recognize and respond appropriately to emotional cues from consumers, thereby creating a more engaging and satisfying experience. In addition, live-streaming platforms should solicit feedback on consumer interactions with AI-based streamers, identifying potential expectancy violations as they occur. For example, platforms can implement a real-time sentiment analysis system that monitors consumer comments and reactions during live streaming. When such sentiment analysis detects potential expectancy violations—such as consumers expressing confusion over a product’s features, dissatisfaction with the virtual streamer’s response time, or frustration with a lack of empathy in communication—managers can then quickly assess the situation, determine whether an expectancy violation has indeed occurred, and take appropriate corrective actions.

Finally, marketers and IT managers can build a comprehensive management mechanism to minimize the negative feelings that consumers may experience (such as distrust and dissatisfaction) and adverse consumer behaviors that can result from expectation violations. For example, they can establish a dedicated customer feedback loop that integrates AI-driven analytics with human oversight, allowing consumers to voice their concerns and feedback through multiple channels while enabling the AI systems to identify negative comments and potential expectancy violations. In addition, marketers could offer appropriate compensation or incentives to affected consumers to mitigate the negative impacts of expectancy violations and strengthen consumer loyalty.

### 6.4. Limitations and Future Research

This study had several limitations, which suggest directions for future research. First, the sample of the current study was limited to Chinese consumers who had previously interacted with virtual streamers. Thus, the results are limited to a single cultural background and may not be directly applicable to other cultural contexts. Future studies could expand the scope of the research by including samples from various countries and cultural contexts to improve the generalizability of our findings.

Second, our study relied on self-reported survey data collected from consumers regarding their actual feelings and experiences with virtual streamers. While this study showed that CMB was not a significant concern, we did not completely exclude the possibility of its existence. Future research could incorporate in-depth interviews, semantic analysis, or experimental methods to provide a more accurate picture of consumers’ experiences and behaviors in the context of virtual streamers’ live-streaming commerce.

Third, our study validated the complex relationship between expectation violations, distrust, dissatisfaction, and discontinuance behavior. However, the reality may be even more complex, with potential moderating factors that could influence the strength and direction of these relationships. For example, personality attributes, such as an affinity for human–computer interactions [5] and moral licensing [37], may moderate the impacts of expectation violations on distrust and dissatisfaction. In addition, there may be some possible interactions between product types (e.g., hedonic products and utilitarian products) and expectations regarding virtual streamer competencies [23]. The impact of cultural differences on consumer expectations and perceptions of virtual streamers remains an open question [77]. Therefore, future research should explore additional moderating factors, such as personality traits, product types, the timing and frequency of interactions, and cultural differences, to validate the robustness of the study or extend the theoretical model.

Finally, although our study presented a rigorous analysis of the phenomenon of virtual streamers in live-streaming commerce, the broader implications of our findings across other domains where digital human interactions are becoming increasingly prevalent have yet to be fully explored [3]. Future research should explore the generalizability to different cultural settings and diverse industry applications, such as AI-driven customer service, virtual healthcare providers, and digital education platforms. Additionally, as AI technology continues to evolve, it is crucial to examine how advancements in natural language-processing and machine learning algorithms might further shape consumers’ expectations and experiences with virtual streamers. By doing so, researchers can uncover new dimensions of consumers’ expectation violations and contribute to the development of more effective strategies for managing consumers’ trust and satisfaction in human–AI interactions.

## Figures and Tables

**Table 1:** Questionnaire items.

Variables	Operational Definition	Measurement Items	Sources
Responsiveness expectation violation (REV)	The consumer’s perception that the virtual streamer fails to meet the consumer’s expectations in assisting them and providing timely service [58,59]	The virtual streamer fails to respond promptly to my questions and requests.	Gao et al. [1]
		This virtual streamer cannot promptly provide relevant information for my inquiry.	
		The responses from the virtual streamer are not relevant to my problems and requests.	
		The virtual streamer lacked enthusiasm in communicating with me.	
Empathy expectation violation (EEV)	The consumer’s perception that the virtual streamer fails to meet the consumer’s expectations in sensing and reacting to the consumer’s thoughts, feelings, and experiences [32]	The virtual streamer provided me with less individual attention than expected.	Yang et al. [32]
		The virtual streamer seldom deals with me in a caring fashion.	
		The virtual streamer does not always have my best interest at heart.	
Professionalism expectation violation (PEV)	The consumer’s perception that the virtual streamer fails to meet the consumer’s expectations in effectively communicating appropriate knowledge, experience, and skills, as well as demonstrating the level of professionalism they should possess in live streaming or selling related products [50,51]	The virtual streamer lacks professional expertise in effectively promoting sales through live streaming.	Li et al. [50] and Guo et al. [30]
		The virtual streamer has limited experience in both live streaming and sales.	
		The virtual streamer is not well-informed about the product’s performance, usage methods, and related knowledge.	
		The virtual streamer offers less information about the products than I expected.	
Distrust (DIT)	The degree to which a consumer believes, with feelings of relative certainty, that the virtual streamer does not have characteristics beneficial to him or her [43]	I am skeptical whether the virtual streamer will do its best to help me if I require help.	Yang et al. [32]
		I am skeptical about whether the virtual streamer is trustworthy.	
		I am worried whether the virtual streamer will be truthful in its dealings with me.	
Dissatisfaction (DIA)	The negative feelings that a consumer may have toward such an overall experience with the virtual streamer [41,46]	I feel dissatisfied about my overall experience of dealing with the virtual streamer.	Mostafa et al. [64]
		I feel displeased about my overall experience of dealing with the virtual streamer.	
		I am not delighted about my overall experience of dealing with the virtual streamer.	
		I feel discontented about my overall experience of dealing with the virtual streamer.	
Discontinuance behavior (DIC)	The consumer reduces their level of engagement with the virtual streamer, or temporarily or permanently ceases to watch [41,42]	I have temporarily stopped watching virtual streamer live streaming.	Peng et al. [6]
		I do not plan to stay much longer in the virtual streamer’s live-streaming room.	
		I would like to discontinue subscribing to the virtual streamer’s channel.	

**Table 2:** Demographics of the respondents (N = 307).

Measure	Items	Frequency	Percentage (%)
Gender	Male	141	45.9
	Female	166	54.1
Age	18–25	95	30.9
	26–35	145	47.2
	36–45	51	16.6
	≥46	16	5.2
Education	High school or less	12	3.9
	Junior college/Undergraduate	253	82.4
	Master or above	42	13.7
Monthly income	<3000 RMB	52	16.9
	3001–6000 RMB	50	16.3
	6001–9000 RMB	87	28.3
	9001–12,000 RMB	62	20.2
	>12,000 RMB	56	18.2

**Table 3:** Common method bias analysis.

Construct	Indicator	Substantive Factor Loading (R1)	R1^2^	Method Factor Loading (R2)	R2^2^
Professionalism expectation violation (PEV)	PEV1	0.780 ***	0.608	0.097	0.009
	PEV2	0.866 ***	0.750	−0.036	0.001
	PEV3	0.937 ***	0.878	−0.202 **	0.041
	PEV4	0.764 ***	0.584	0.114	0.013
Empathy expectation violation (EEV)	EEV1	0.810 ***	0.656	0.112 *	0.013
	EEV2	0.887 ***	0.787	0.003	0.000
	EEV3	0.923 ***	0.852	−0.121 *	0.015
Responsiveness expectation violation (REV)	REV1	0.897 ***	0.805	−0.032	0.001
	REV2	0.889 ***	0.790	−0.021	0.000
	REV3	0.758 ***	0.575	0.098	0.010
	REV4	0.855 ***	0.731	−0.046	0.002
Distrust (DIT)	DIT1	0.857 ***	0.734	0.012	0.000
	DIT2	0.833 ***	0.694	0.087	0.008
	DIT3	0.945 ***	0.893	−0.105 *	0.011
Dissatisfaction (DIA)	DIA1	0.808 ***	0.653	0.062	0.004
	DIA2	0.927 ***	0.859	−0.090	0.008
	DIA3	0.757 ***	0.573	0.132 *	0.017
	DIA4	0.966 ***	0.933	−0.109	0.012
Discontinuance behavior (DIC)	DIC1	0.915 ***	0.837	−0.051	0.003
	DIC2	0.813 ***	0.661	0.091	0.008
	DIC3	0.911 ***	0.830	−0.042	0.002
Average		0.862	0.747	−0.002	0.008
Ratio		88.35			

Note: *** *p* < 0.001; ** *p* < 0.01; * *p* < 0.05.

**Table 4:** Reliability and validity analysis.

Construct	Items	Loading	VIF	Cronbach’s α	CR	AVE
Professionalism expectation violation (PEV)	PEV1	0.864 ***	2.264	0.851	0.864	0.692
	PEV2	0.833 ***	2.202			
	PEV3	0.763 ***	1.924			
	PEV4	0.863 ***	2.116			
Empathy expectation violation (EEV)	EEV1	0.902 ***	2.332	0.867	0.874	0.789
	EEV2	0.892 ***	2.256			
	EEV3	0.870 ***	2.182			
Responsiveness expectation violation (REV)	REV1	0.871 ***	2.544	0.872	0.874	0.722
	REV2	0.870 ***	2.539			
	REV3	0.843 ***	2.009			
	REV4	0.814 ***	1.894			
Distrust (DIT)	DIT1	0.868 ***	1.995	0.849	0.855	0.768
	DIT2	0.906 ***	2.395			
	DIT3	0.854 ***	1.971			
Dissatisfaction (DIA)	DIA1	0.865 ***	2.282	0.886	0.887	0.745
	DIA2	0.845 ***	2.139			
	DIA3	0.876 ***	2.427			
	DIA4	0.867 ***	2.370			
Discontinuance behavior (DIC)	DIC1	0.869 ***	2.085	0.853	0.858	0.773
	DIC2	0.891 ***	2.119			
	DIC3	0.878 ***	2.109			

Note: *** *p* < 0.001.

**Table 5:** Discriminant validity analysis (Fornell–Larcker and HTMT).

	DIA	DIC	DIT	EEV	PEV	REV
DIA	**0.863**	*0.775*	*0.741*	*0.683*	*0.730*	*0.718*
DIC	0.676	**0.879**	*0.689*	*0.605*	*0.580*	*0.549*
DIT	0.647	0.590	**0.876**	*0.661*	*0.681*	*0.639*
EEV	0.602	0.524	0.571	**0.888**	*0.552*	*0.672*
PEV	0.644	0.500	0.582	0.486	**0.832**	*0.696*
REV	0.632	0.475	0.552	0.587	0.606	**0.850**

Note: The bold diagonal elements signify the square root of the AVE for each construct. The hetero-trait–mono-trait ratio is indicated in italics.

**Table 6:** Mediating effect results.

Path	Indirect Effect	t-Values	Bootstrap 95% CI		Mediation
			LLCI	ULCI	
PEV→DIT→DIC	0.086	3.422 **	0.044	0.144	Yes
EEV→DIT→DIC	0.082	2.817 **	0.036	0.151	Yes
REV→DIT→DIC	0.044	2.238 *	0.014	0.093	Yes
PEV→DIA→DIC	0.180	4.552 ***	0.109	0.263	Yes
EEV→DIA→DIC	0.143	3.766 ***	0.078	0.227	Yes
REV→DIA→DIC	0.128	3.615 ***	0.061	0.199	Yes

Note: *** *p* < 0.001; ** *p* < 0.01; * *p* < 0.1.

## Data Availability

The raw data supporting the conclusions of this article are available upon request from the corresponding author.
